# Predicted Release and Analysis of Novel ACE-I, Renin, and DPP-IV Inhibitory Peptides from Common Oat (*Avena sativa*) Protein Hydrolysates Using in Silico Analysis

**DOI:** 10.3390/foods6120108

**Published:** 2017-12-04

**Authors:** Stephen Bleakley, Maria Hayes, Nora O’ Shea, Eimear Gallagher, Tomas Lafarga

**Affiliations:** 1 Food Biosciences Department, Teagasc Food Research Centre, Ashtown, D15 Dublin, Ireland; Stephen.Bleakley@teagasc.ie; 2 School of Biological Sciences, College of Sciences and Health and Environment, Sustainability and Health Institute, DIT Kevin Street, D08 NF82 Dublin, Ireland; 3 Food Chemistry and Technology Department, Teagasc Food Research Centre, Moorepark, Fermoy, Co. Cork P61 C996, Ireland; Norah.O’Shea@teagasc.ie; 4 Food Quality and Sensory Science Department, Teagasc Food Research Centre, Ashtown, Dublin 15, Ireland; Eimear.Gallagher@teagasc.ie; 5 Parc Científic I Tecnològic Agroalimentari de Lleida, Parc de Gardeny, Edifici Fruit Centre, Institut de Recerca, Tecnològia Agroalimentàries (IRTA), 25003 Lleida, Spain; tomas.lafarga@irta.cat

**Keywords:** oats, *Avena sativa*, bioactive peptides, ACE-I, renin, DPP-IV, renin-angiotensin-aldosterone system

## Abstract

The renin-angiotensin-aldosterone system (RAAS) plays an important role in regulating hypertension by controlling vasoconstriction and intravascular fluid volume. RAAS itself is largely regulated by the actions of renin (EC 3.4.23.15) and the angiotensin-I-converting enzyme (ACE-I; EC 3.4.15.1). The enzyme dipeptidyl peptidase-IV (DPP-IV; EC 3.4.14.5) also plays a role in the development of type-2 diabetes. The inhibition of the renin, ACE-I, and DPP-IV enzymes has therefore become a key therapeutic target for the treatment of hypertension and diabetes. The aim of this study was to assess the bioactivity of different oat (*Avena sativa*) protein isolates and their ability to inhibit the renin, ACE-I, and DPP-IV enzymes. In silico analysis was carried out to predictthe likelihood of bioactive inhibitory peptides occurring from oat protein hydrolysates following in silico hydrolysis with the proteases papain and ficin. Nine peptides, including FFG, IFFFL, PFL, WWK, WCY, FPIL, CPA, FLLA, and FEPL were subsequently chemically synthesised, and their bioactivities were confirmed using in vitro bioassays. The isolated oat proteins derived from seven different oat varieties were found to inhibit the ACE-I enzyme by between 86.5 ± 10.7% and 96.5 ± 25.8%, renin by between 40.5 ± 21.5% and 70.9 ± 7.6%, and DPP-IV by between 3.7 ± 3.9% and 46.2 ± 28.8%. The activity of the synthesised peptides was also determined.

## 1. Introduction

High blood pressure is the single largest risk factor attributed to deaths worldwide. It is responsible for 12.8% of deaths, and affects all countries and income groups [1]. Furthermore, high systolic blood pressure is globally attributable to 51% of strokes, 45% of ischaemic heart disease, and between 37% (Southeast Asia) and 54% (European countries) of cardiovascular deaths [1]. Hypertension is therefore a considerable problem in our society, not only placing a great burden on our healthcare system, but also having a substantial impact on the economy, with direct medical costs of cardiovascular disease (CVD) estimated to increase three-fold from $273 billion in 2010 to $818 billion in 2030 in the United States alone [2]. The renin-angiotensin-aldosterone system (RAAS) plays an important role in regulating blood pressure by controlling arteriolar vasoconstriction and intravascular fluid volume. RAAS itself is largely regulated by the actions of the enzyme angiotensin-I-converting enzyme (ACE-I; EC 3.4.15.1), which increases blood pressure [3]. 

ACE-I inhibitors have therefore become one of the most commonly studied drugs, with global annual sales exceeding $6 billion USD. ACE-I inhibitory drugs can be considered one of the major protease inhibitor success stories [4]. Synthetic ACE-I inhibitor drugs such as captopril, enalapril, and alacepril, often come with several side effects, including hypotension, dry cough, and impaired renal function [5]. Functional foods with antihypertensive bioactivities have therefore become a popular alternative to synthetic drugs, especially for individuals who are borderline hypertensive and do not warrant the prescription of pharmaceutical drugs [6].

Gliptins or dipeptidyl peptidase IV (DPP-IV) inhibitors can block the action of the enzyme DPP-IV, and may be used to treat diabetes mellitus type 2. DPP-IV inhibitors increase incretin levels, inhibit glucagon release, increase insulin secretions, and decrease gastric emptying and blood glucose levels.

In addition to providing energy and amino acids essential for growth, it has become increasingly recognised that some dietary proteins contain biologically active peptides that can impart a beneficial physiological effect [7]. Such bioactive peptides are typically 2–30 amino acids in length, and can exert their physiological response as a result of their hormone-like properties or by acting as antagonistic receptor inhibitors [8]. Bioactive peptides are inactive within the precursor protein, and can be released through hydrolysis by digestive enzymes or fermentation. The function of these peptides covers a variety of activities, including antihypertensive [9], hypoglycemic [10], antiamnestic [11], antimicrobial [12], antithrombotic [13], antioxidative [14], hypocholesterolemic [15], gastrointestinal absorption modulation [16], appetite suppression [17], opioids [18], immunomodulation [19], and cytomodulation [20].

Milk proteins remain the most common food resource for bioactive peptide generation [7,21]. The most common ACE-I inhibitory peptides, Ile-Pro-Pro and Val-Pro-Pro, are derived from casein in milk [9]. However, bioactive peptides have also been identified in a number of plant and animal protein sources, including rice [22], soybean [23], wheat [24], seaweed [25], pea [26], broccoli [27], garlic [28], egg [29], meat [30], blood [31] and fish [32]. 

The common oat (*Avena sativa*) is a promising source of bioactive peptides, with several peptides already identified, but it has not been fully explored yet [33]. This study isolated proteins from seven different oat varieties, and identified the bioactivities of these extracts. In addition, it identified ACE-I, renin (EC 3.4.23.15), and DPP-IV inhibitory peptides following in silico digestion of common oat proteins. Selected peptides were synthesised and tested for their ability to inhibit ACE-I, renin, and DPP-IV.

## 2. Materials and Methods 

### 2.1. Materials

Seven strains of de-hulled and milled oats were used for protein extraction, including Barra, Husky, Maesbro, Mascan, Rhapsody, Selwyn, and Vodka. The oat samples were harvested in 2014/2015 by John Finnan, Teagasc Oakpark, and were also supplied by the Tillage manager at Glanbia PLC (Glanbia PLC, Kilkenny, Ireland). The naked, de-husked grains were used to obtain a higher yield, as they are significantly higher in protein and lower in crude fiber [34]. Ammonium sulfate was obtained from Sigma Aldrich (Wicklow, Ireland). 

### 2.2. Oat Protein Extraction 

A 2% (*w*/*v*) solution of milled oats in distilled deionised water (ddH_2_O) was sonicated using an Ultrasonic Bath Branson 3510 at 42 kHz for 1 h. This was followed by stirring overnight at 4 °C. The oats were then centrifuged at 10,000× *g* for 1 h at 4 °C using a Sorvall™ Lynx 6000 centrifuge (Thermo Scientific™, Dublin, Ireland). The supernatant was then collected and stored at 4 °C, while the pellet was re-suspended in 4% (*w*/*v*) ddH_2_O. The resuspended oats were sonicated for 1 h, stirred overnight at 4 °C, and centrifuged at 10,000× *g* for 1 h at 4 °C. The supernatant was then pooled with the first supernatant for further processing. An 80% ammonium sulfate-saturated solution was made with the pooled oat supernatant, and stirred for 1 h at 4 °C, followed by centrifugation at 17,000× *g* for 1 h at 4 °C. The pellet was resuspended and dialysed overnight at 4 °C. Samples were stored at −20 °C and subsequently freeze-dried using an industrial-scale freeze drier, FD 80 model (Cuddon Engineering, Marlborough, New Zealand). 

### 2.3. In Silico Digestion

**Figure 1:**
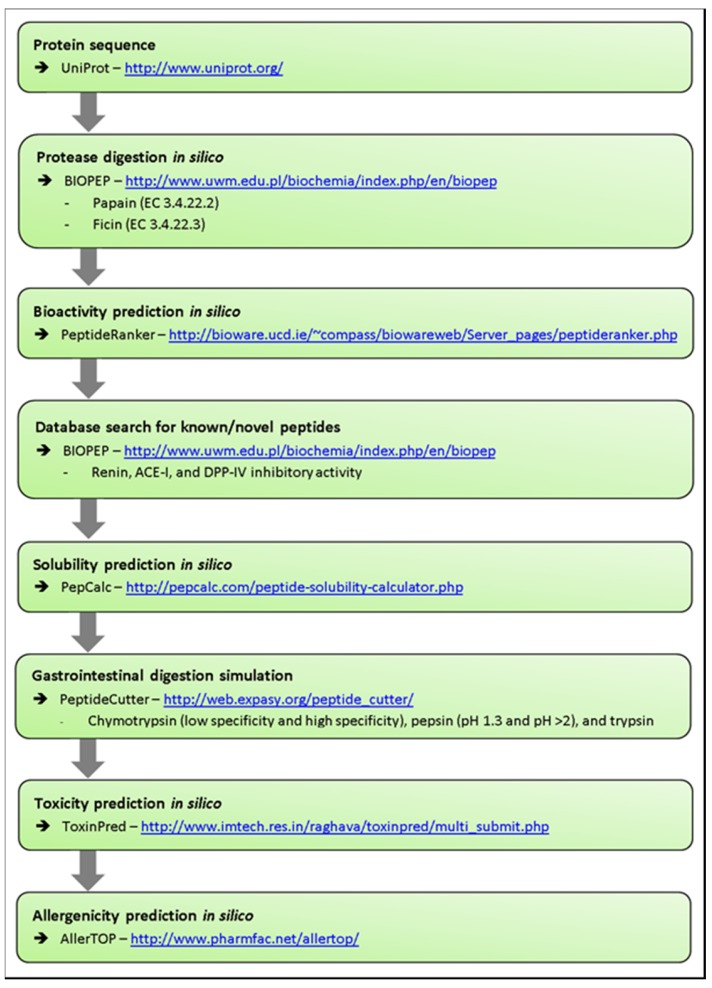
Methodology for in silico digestion and bioactivity prediction of oat protein hydrolysates.

The primary proteins found in oats were identified from the literature, with protein sequences obtained from the UniProt database, available at http://www.uniprot.org (Table 1). Each protein sequence was digested in silico with papain (EC 3.4.22.2) or ficin (EC 3.4.22.3) using BIOPEP, which is available at http://www.uwm.edu.pl/biochemia/index.php/en/biopep [35], and the method shown in Figure 1.

### 2.4. Bioactivity Prediction In Silico

The peptides that resulted from oat protein hydrolysates were ranked for bioactivity according to their PeptideRanker score and known inhibitory peptide characteristics (Table 2), as previously described [36]. PeptideRanker, available at http://bioware.ucd.ie/~compass/biowareweb/Server_pages/peptideranker.php [37], is a server that predicts how likely a peptide is to be bioactive based on an N-to-1 neural network algorithm [37]. PeptideRanker predicts how likely peptides are to be bioactive, but does not indicate the targets for which they are most suitable. A literature search was therefore carried out to identify the characteristics of peptides that have been shown to increase the likelihood of inhibition with the enzyme targets in this study (Table 2). 

Additional in silico analysis was carried out to predict water solubility, resistance to gastrointestinal digestion, toxicity, and allergenicity (Figure 1). Solubility in water was predicted using PepCalc, which is available at http://pepcalc.com. Resistance to digestion was predicted using PeptideCutter, which is available at http://web.expasy.org/peptide_cutter/ [36] with the enzymes’ chymotrypsin-low specificity, chymotrypsin-high specificity, pepsin (pH 1.3), pepsin (pH > 2), and trypsin. Toxicity was scanned with default settings using ToxinPred, which is available at http://www.imtech.res.in/raghava/toxinpred/multi_submit.php [36]. Allergenicity was predicted using AllerTOP, which is available at http://www.pharmfac.net/allertop/ [36] (Figure 1). 

### 2.5. Chemical Synthesis of Peptides

The selected peptides (FFG, IFFFL, PFL, WWK, WCY, FPIL, CPA, FLLA, FEPL) were synthesised by microwave-assisted solid phase peptide synthesis (MW-SPPS) performed on a Liberty microwave peptide synthesiser (Mathews, NC, USA). Peptides were synthesised on H-Ala-HMPB-ChemMatrix and H-Ile-HMPB-ChemMatrix resins (PCAS Biomatrix Inc., Quebec, QC, Canada) and purified using RP-HPLC on a Semi Preparative Jupiter Proteo (4u, 90A) column (Phenomenex, Cheshire, UK). Fractions containing the desired molecular mass were identified using matrix-assisted laser desorption ionisation time-of-flight mass spectrometry (MALDI-TOFMS) and were pooled and freeze-dried on a Genevac HT 4X lyophilizer (Genevac Ltd., Ipswich, UK).

### 2.6. Renin Inhibition Assay

Protein isolates from all seven oat varieties and selected synthesised peptides were tested in vitro for renin inhibition activity. The Renin Inhibition Screening Assay (Cambridge BioSciences, Cambridge, UK) was carried out as per the manufacturer’s instructions. Briefly, 10 μL of each sample inhibitor at a concentration of 1 mg/mL dimethyl sulfoxide (DMSO) was added to 20 μL renin substrate, 150 μL assay buffer, and 10 μL renin, in triplicate. The samples were incubated at 37 °C for 15 min, and read with excitation wavelengths of 340 nm and emission wavelengths of 500 nm. Fluorescence was read using a FLUOstar Omega microplate reader (BMG LABTECH GmbH, Offenburg, Germany). The percentage inhibition was calculated using the following equation:

% Renin inhibition = 100% Initial activity − Inhibitor × 100/100% Initial activity (1)

### 2.7. ACE-I Inhibition Assay

Protein isolates from all seven oat varieties and selected synthesised peptides were tested in vitro for ACE-I inhibition. The bioassay (ACE Kit—WST, Dojindo Laboratories, Kumamoto, Japan) was carried out according to the manufacturer’s instructions. First, 20 μL of each sample inhibitor at a concentration of 1 mg/mL ddH_2_O was added to 20 μL substrate and 20 μL enzyme working solution in triplicate. Samples were incubated at 37 °C for 1 h. Each well then had 200 μL indicator working solution added, followed by a further incubation at room temperature for 10 min. Absorbance at 450 nm was read using a FLUOstar Omega microplate reader (BMG LABTECH GmbH, Offenburg, Germany). The percentage inhibition was calculated using the following equation:

% ACE-I inhibition = 100% Initial activity − Inhibitor × 100/100% Initial activity(2)

### 2.8. DPP-IV Inhibition Assay

Protein isolates from all seven oat varieties and selected synthesised peptides were tested in vitro for DPP-IV inhibition. The bioassay (DPP-IV Inhibitor Screening Assay Kit, Cayman Chemical, Ann Arbor, MI, USA) was carried out as per the manufacturer’s instructions. First, 10 μL of each sample inhibitor at a concentration of 1 mg/mL assay buffer was added to 30 μL diluted assay buffer, 10 μL diluted DPP-IV, and 50 μL substrate solution, in triplicate. Samples were incubated at 37 °C for 30 min. Fluorescence was read with excitation wavelengths of 355 nm and emission wavelengths of 460 nm using a FLUOstar Omega microplate reader (BMG LABTECH GmbH, Offenburg, Germany). The percentage inhibition was calculated using the following equation:

% DPP-IV inhibition = 100% Initial activity − Inhibitor × 100/100% Initial activity (3)

## 3. Results

### 3.1. In Silico Bioactivity Prediction

In silico analysis of oat protein isolates identified a number of bioactive peptides that had previously been reported in the BIOPEP database (Table 3). These peptides were not selected for chemical synthesis.

Based on the known characteristics of renin, ACE-I and DPP-IV inhibitory peptides (Table 2), novel peptides were identified for in vitro analysis in this study. The peptides chosen had the amino acid sequences FFG, IFFFL, PFL, WWK, WCY, FPIL, CPA, FLLA, and FEPL (Table 4). The tables predicted to have the greatest bioactivities are shown in Table A1 (Appendix).

Bioactive peptides need to survive degradation by gastrointestinal digestion, reach their target intact, and maintain bioavailability in order to exert a beneficial physiological effect [44]. The novel peptides were therefore analysed in silico for predicted solubility, resistance to digestion, toxicity, and allergenicity (Table 4). Most of the peptides were expected to be poorly soluble due to their high hydrophobic residue content. Most of the peptides were also expected to be broken down by gastrointestinal digestive enzymes, although this could be overcome by methods such as encapsulation [45]. The peptides were also expected to be non-toxic and non-allergenic (Table 4).

### 3.2. Renin Inhibition

**Figure 2:**
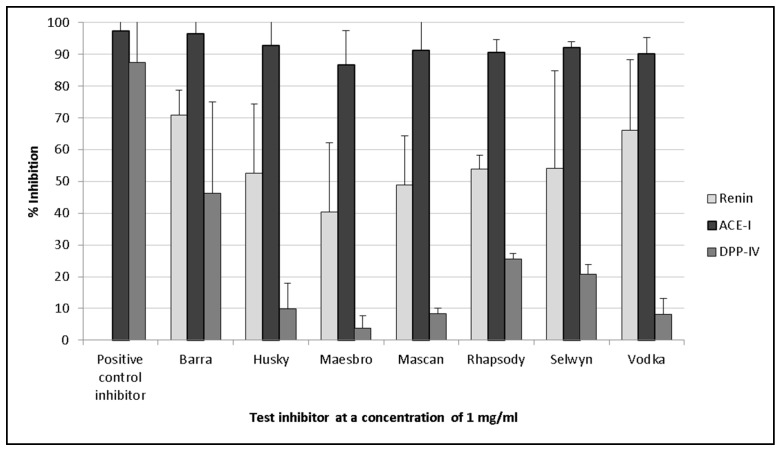
Inhibition bioactivity for the targets renin, angiotensin-I-converting enzyme (ACE-I), and dipeptidyl peptidase-IV (DPP-IV) using protein isolates from seven oat varieties at a test concentration of 1 mg of extract (dry weight)/mL. Values are the mean of triplicate samples. Captopril was used as the positive control for ACE-I inhibition, while sitagliptin was used as the positive control for DPP-IV inhibition.

Oat protein isolates displayed renin inhibition values ranging between 40.5% (±2.16%) and 70.9% (±7.7%) (Figure 2). Barra oat had the highest levels of renin inhibition, with 70.9% (±7.7%) inhibition, followed by Vodka oat, which had 66.1% (±22.1%) inhibition. There was significantly lower renin inhibition activity from synthesised peptides compared with that of the oat protein isolates. 

However, renin inhibition was not observed with the synthesised peptides. Only the peptide IFFFL was found to inhibit renin by 17.1% (±2.6%) when tested at a concentration of 1 mg/mL compared with the control.

### 3.3. ACE-I Inhibition

The crude oat protein extracts inhibited ACE-I by between 86.6% (±10.7%) and 96.5% (±25.8%). These results were therefore comparable with that of the positive control captopril, which was found to inhibit ACE-I by 97.7% (±23.2%) when tested at a concentration of 1 mg/mL (Figure 2). Protein extracted from Barra oat varieties displayed the highest ACE-I inhibition values, and inhibited the enzyme by 96.5% (±25.8%) when assayed at a concentration of 1 mg/mL compared with the positive control.

**Figure 3:**
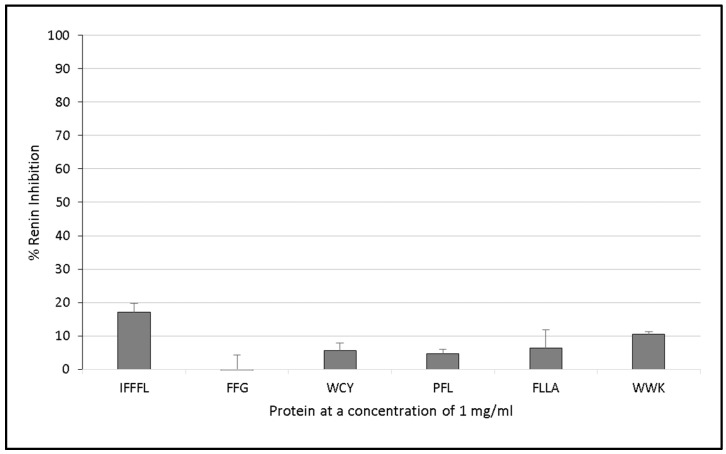
Renin inhibition assay of several chemically synthesised peptides from predicted oat protein hydrolysates following in silico digestion with the proteases papain and ficin. Peptides were assayed at a concentration of 1 mg protein/mL. Values are the mean of triplicate samples.

The highest levels of ACE-I inhibition with the synthesised peptides were observed for the peptides WCY (97.8 ± 21.7%), FLLA (97 ± 16.2%), and WWK (95.3 ± 14.2%) (Figure 3). The lowest levels of inhibition were seen with IFFFL (53 ± 41.2%) and FEPL (48.9 ± 7.8%), contradicting previous findings of the beneficial effect of leucine at the *C*-terminus of an ACE-I inhibiting bioactive peptide [40]. However, the poor activity from IFFFL and FEPL could also be due to the larger peptide size as is the case with the peptide PFL, which also has a C-terminal leucine, and displayed higher levels of ACE-I inhibition (81.4 ± 33.8%). Alternatively, the lower ACE-I inhibitory values observed for FEPL could also be due to the presence of proline at the penultimate position within the peptide, which has been suggested to reduce the binding affinity of peptides with the ACE-I enzyme [46].

### 3.4. DPP-IV Inhibition

The protein isolates of the seven oat varieties did not display significant DPP-IV inhibitory activities when assayed at a concentration of 1 mg/mL, and values ranged from 3.7% (±3.9%) to 46.3% (±8.8%) compared with the positive control sitagliptin (87.4 ± 4%) (Figure 2). The highest DPP-IV inhibition activity was seen with the protein extracts of Barra oat (46.3 ± 8.8%), Rhapsody oat (25.4 ± 1.8%), and Selwyn oat (20.7 ± 3.1%). 

Peptides containing the sequence Xaa-Pro (where Xaa represents any amino acid, and proline is present at the second residue from the *N*-terminus) have been found to be effective DPP-IV inhibitors [43]. The peptides CPA (22.2 ± 4.8%) and FPIL (13.1 ± 3.2%) were the only chemically synthesised peptides that were found to inhibit DPP-IV at a concentration of 1 mg/mL. 

## 4. Discussion

Functional foods in the form of bioactive peptides offer additional physiological benefits beyond their basic nutritional value. Bioactive peptides that inhibit the enzymes within RAAS, such as renin and ACE-I, are used as alternatives to antihypertensive pharmaceutical drugs [7]. Similarly, DPP-IV inhibitory bioactive peptides have also been shown to effectively prevent the onset of type-2 diabetes by preventing the cleavage of the glucagon-like peptide 1 (GLP-1) and glucose-dependent insulinotropic peptide (GIP) incretins [43]. Bioactive peptides have been identified from a variety of food sources, including milk proteins, seaweed, and meat [21,28,30], as well as a number of grains, including rice, soybean, wheat, and barley [22,23,24,54]. This study determined the ACE-I, renin, and DPP-IV inhibitory activities of oat protein isolates and peptides synthesised in vitro.

While there are pharmaceutical therapies for hypertension and type-2 diabetes, they are often accompanied by adverse side effects, such as a dry cough, anaphylaxis, renal impairment, hyperkalaemia, and inflammation-related pain [55,56,57]. Pharmaceuticals have several reported side effects, but are active at lower concentrations than food-derived bioactive peptides. However, peptides consumed with IC_50_ values of 100–500 μM have been shown to be active in vivo and inhibit ACE-I, renin, and DPP-IV enzymes [58,59]. Furthermore, functional foods with bioactivities can be a beneficial alternative to synthetic drugs for individuals who have borderline disease states and do not warrant the prescription of pharmaceutical drugs [6]. 

Oat protein isolates were found to inhibit renin by between 40.5% (±2.16%) and 70.9% (±7.7%) (Figure 2) in vitro when assayed at a concentration of 1 mg/mL protein, but the selected synthesised peptides did not inhibit renin to the same degree (Figure 3). This demonstrates that it is necessary to carry out in vitro work and characterise all of the peptides present in a hydrolysate. The observed activities could also be due to other compounds present in the protein isolate, such as phenolic compounds, and phenolic compounds may still be present despite the use of dialysis to concentrate the protein fraction. The active peptide(s) could have been previously identified (Table 4) or were not chosen for synthesis. The characteristics of renin inhibitory peptides are not as well defined as other bioactive peptide targets (such as ACE-I inhibitory peptides, for example) due to the notably poor potency that has been observed with renin peptide inhibitors [60]. The peptides IFFFL, FLLA, and WWK (Figure 3) were chosen for chemical synthesis based on previously published literature by Udenigwe and colleagues (2012), who observed that the bulky amino acids at the *N*-terminus aided in renin inhibition (Table 3). 

**Figure 4:**
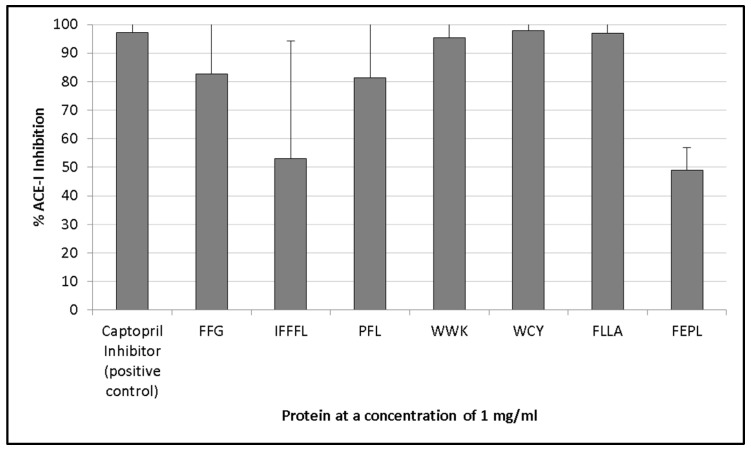
ACE-I inhibition assay of several synthesised peptides that were identified in silico from the digestion of oat proteins with papain or ficin, which are both proteases. The activity of peptides was compared with that of a captopril inhibitor (positive control). Peptides were assayed at a concentration of 1 mg protein of extract/mL. Values are the mean of triplicate samples.

The oat protein isolates generated in this work were all found to inhibit the enzyme ACE-I by between 86.6% (±10.7%) and 96.5% (±25.8%) (Figure 2). This was significantly greater than a similar study assessing crude protein extracts derived from barley [54]. Unlike the renin assay, there were comparable levels of activity in the selected synthesised peptides and the oat protein extracts (Figure 4). This suggests that the correct peptides were selected for chemical synthesis, which is largely because the mechanism of the action of ACE-I inhibitory peptides is better understood. 

**Figure 5:**
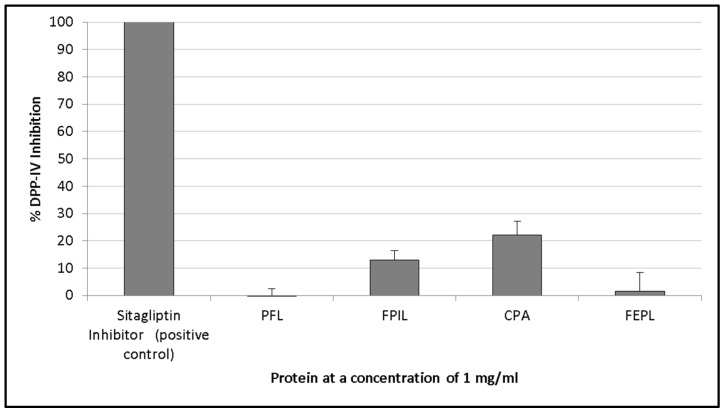
DPP-IV inhibition of several synthesised peptides that were identified in silico from the hydrolysis of oat proteins with papain or ficin, which are both proteases. The activity of peptides was compared with that of a sitagliptin inhibitor (positive control). Peptides were assayed at a concentration of 1 mg/mL. Values are the mean of triplicate samples.

The Barra oat protein isolate had the highest activity with 46.3% (±28.8%) inhibition, followed by Rhapsody oat (25.4 ± 1.8%) and Selwyn oat (20.7 ± 3.1%). Similarly, the selected synthesised peptides also had relatively poor DPP-IV inhibition (Figure 5). DPP-IV is a proline-specific endopeptidase that cleaves dipeptides from the *N*-terminus [61]. Of the nine peptides synthesised (Table 4), those containing a proline residue were therefore selected for testing DPP-IV inhibition.

In silico methods were used heavily in this study to evaluate the potential of oat protein to generate bioactive peptides, as well as predict generated bioactive peptides following hydrolysis with the food-grade proteases papain and ficin. Similar bioinformatic techniques have already been described in other studies [36], which highlighted their value in reducing time and expense for the preliminary screening of novel sources of bioactive peptides. The preparatory in silico screening of potential bioactive peptides for characteristics such as allergenicity, toxicity, solubility, and degradation are additional important uses for such online software and tools, especially when presenting these peptides for possible human consumption. 

## 5. Conclusions

There has been little work carried out to evaluate the potential of the common oat (*Avena sativa*) as a potential source of bioactive peptides. Bioinformatics techniques were used to predict whether oats were indeed a rich source of bioactive peptides following in silico hydrolysis with the proteases papain and ficin on the main storage proteins. Oat protein isolates displayed the highest inhibition bioactivity against the target ACE-I (86.6–96.5%), with lower inhibition levels observed with renin (40.5–70.9%) and DPP-IV (3.7–46.3%). Following the chemical synthesis of nine novel peptides, in vitro bioassays gave mixed results as to their efficacy in inhibiting the injurious enzyme targets ACE-I (48.9–97.8%), renin (0–17.1%), and DPP-IV (0–22.2%). The in silico methods utilised in this study correctly identified ACE-I inhibitory peptides, beyond that of renin and DPP-IV inhibitory peptides. This could reflect a more specific use of these in silico methods until the characteristics of renin and DPP-IV inhibitory peptides are better understood.

## Figures and Tables

**Table 1:** The main storage proteins found in oats.

Protein	UniProt ID	Sequence **	Amino Acid Length	Molecular Mass (Da)
**11S globulin**	Q38780	MATTSFPSMLFYFCIFLLFHGSMAQLFGQSSTPWQSSRQGGLRGCRFDRLQAFEPLRQVRSQAGITEYFDEQNEQFRCTGVSVIRRVIEPQGLVLPQYHNAPALVYILQGRGFTGLTFPGCPATFQQQFQPFDQSQFAQGQRQSQTIKDEHQRVQRFKQGDVVALPAGIVHWCYNDGDAPIVAIYVFDVNNNANQLEPRQKEFLLAGNNKREQQSGNNIFSGLSVQLLSEALGISQQAAQRIQSQNDQRGEIIRVSQGLQFLKPIVSQQVPGEQQVYQPIQTQEGQATQYQVGQSTQYQVGKSTPYQGGQSSQYQAGQSWDQSFNGLEENFCSLEARKNIENPQHADTYNPRAGRITRLNSKNFPILNIVQMSATRVNLYQNAILSPFWNINAHSVIYMIQGHARVQVVNNNGQTVFNDILRRGQLLIVPQHFVVLKKAEREGCQYISFKTNPNSMVSHIAGKSSILRALPIDVLANAYRISRQEARNLKNNRGEEFGAFTPKLTQKGFQSYQDIEEGSSSPVRASE	527	59,406
**12S globulin**	P12615	MATTRFPSLLFYSCIFLLCNGSMAQLFGQSFTPWQSSRQGGLRGCKFDRLQAFEPLRQVRSQAGITEYFDEQNEQFRCAGVSVIRRVIEPQGLLLPQYHNAPGLVYILQGRGFTGLTFPGCPATFQQQFQQFDQARFAQGQSKSQNLKDEHQRVHHIKQGDVVALPAGIVHWCYNDGDAPIVAVYVFDVNNNANQLEPRQKEFLLAGNNKREQQFGQNIFSGFSVQLLSEALGISQQAAQKIQSQNDQRGEIIRVSQGLQFLKPFVSQQGPVEHQAYQPIQSQQEQSTQYQVGQSPQYQEGQSTQYQSGQSWDQSFNGLEENFCSLEARQNIENPKRADTYNPRAGRITHLNSKNFPTLNLVQMSATRVNLYQNAILSPYWNINAHSVMHMIQGRARVQVVNNHGQTVFNDILRRGQLLIIPQHYVVLKKAEREGCQYISFKTTPNSMVSYIAGKTSILRALPVDVLANAYRISRQESQNLKNNRGEEFGAFTPKFAQTGSQSYQDEGESSSTEKASE	518	58,545
**Avenin-3**	P80356	MKTFLIFALLAMAATMATAQFDPSEQYQPYPEQQQPILQQQQMLLQQQQQMLLQQQPLLQVLQQQLNPCRQFLVQQCSPVAVVPFLRSQILQQSSCQVMRQQCCRQLEQIPEQLRCPAIHSVVQAIIMQQQQFFQPQMQQQFFQPQMQQVTQGIFQPQMQQVTQGIFQPQLQQVTQGIFQPQMQGQIEGMRAFALQALPAMCDVYVPPHCPVATAPLGGF	220	25,275
**Avenin-E**	Q09114	TTTVQYNPSEQYQPYPEQQEPFVQQQPFVQQQQQPFVQQQQMFLQPLLQQQLNPCKQFLVQQCSPVAVVPFLRSQILRQAICQVARQQCCRQLAQIPEQLRCPAIHSVVQAIILQQQQQQQFFQPQLQQQVFQPQLQQVFNQPQQQAQFEGMRAFALQALPAMCDVYVPPQCPVATAPLGGF	182	21,036
**Avenin-F**	Q09097	TTTVQYDPSEQYQPYPEQQEPFVQQQPPFVQQQQPFVQQQEPF	43	5214
**Avenin-A**	Q09095	PSEQYQPYPEQQQPFLQQQPLELQQQQXXLVLFLQK	36	4393
**Avenin**	P27919	MKIFFFLALLALVVSATFAQYAESDGSYEEVEGSHDRCQQHQMKLDSCREYVAERCTTMRDFPITWPWKWWKGGCEELRNECCQLLGQMPSECRCDAIWRSIQRELGGFFGTQQGLIGKRLKIAKSLPTQSTWALSAISPNSMVSHIAGKSSILRALPVDVLANAYRISRQEARNLKNNRGQESGVFTPKFTQTSFQPYPEGEDESSLINKASE	214	24,230
**Tryptophanin/2S albumin**	A7U440	MKALFLLAFLALAASAAFAQQYADTGVGGWDGCMPEKARLNSCKDYVVERCLTLKDIPITWPWKWWKGGCESEVRSQCCMELNQIAPHCRCKAIWRAVQGELGGFLGFQQSEIMKQVHVAQSLPSRCNMGPNCNFPTNLGYY	142	15,901

** Amino acid nomenclature: A, ala, alanine; C, cys, cysteine; D, asp, aspartic acid; E, glu, glutamic acid; F, phe, phenylalanine; G, gly, glycine; H, his, histidine; I, Ile, isoleucine; K, lys, lysine; L, leu, leucine; M, met, methionine; N, asn, asparagine; P, pro, proline; Q, gln, glutamine; R, arg, arginine; S, ser, serine; T, thr, threonine; V, val, valine; W, trp, tryptophan; Y, tyr; tyrosine; X, undetermined amino acid. Protein sequences were obtained from the UniProt database, which is available at http://www.uniprot.org/.

**Table 2:** Characteristic criteria used to identify tripeptides with predicted renin, angiotensin-I-converting enzyme (ACE-I), and dipeptidyl peptidase-IV (DPP-IV) inhibition activity.

	Location	Group	Amino Acids	Reference
**Renin inhibitory peptide characteristics**	N1/*N*-terminus	Hydrophobic	Ala, Gly, Val, Leu, Ile, Pro, Phe, Met, Trp	[38]
	N2	N/A		
	N3/C-terminus	Bulky	Trp, Val, Ile, Leu, Tyr, Met, Phe,	[38]
**ACE-I inhibitory peptide characteristics**	N1/*N*-terminus	Hydrophobic (Small with low lipophilicity)	Val, Ile, Leu	[39]
	N2	Positively charged (Large with high lipophilicity & low electronic properties)	Leu, Arg	[39]
	N3/*C*-terminus	Aromatic acids (Small with low lipophilicity & high electronic properties)	Pro, Phe, Trp	[39]
		-	Leu	[40]
		Positively charged	Lys, Arg	[41]
		-	Pro	[41]
**DPP-IV inhibitory peptide characteristics**	N1/*N*-terminus	Hydrophobic or aromatic	Leu, Ile, Val, Phe, Trp, Try	[42]
	N2	N/A		
	N3/*C*-terminus	-	Pro, Ala	

**Table 3:** Previously identified peptides from oat (*Avena sativa*) generated using in silico hydrolysis with papain or ficin.

Peptide	Peptide Ranker Score	BIOPEP ID	Activity Description	IC_50_	Mass (Da)	Reference
**FG**	0.99	7605	ACE-I inhibitor	3700.0 μM	222.229	[43,44,45,46]
**PF**	0.99	8854	DPP-IV inhibitor	N/A	262.294	[47]
**FL**	0.99	8555	DPP-IV inhibitor	399.58 μM	278.337	[43]
**FY**	0.98	3556	ACE-I inhibitor	25 μM	328.347	[48]
**FA**	0.96	3176	DPP-IV inhibitor	N/A	236.256	[49]
**FN**	0.95	8778	DPP-IV inhibitor	N/A	279.281	[47]
**MG**	0.94	7609	ACE-I inhibitor	4800 μM	206.25	[46]
**PG**	0.88	7625	ACE-I inhibitor	17,000 μM	172.169	[46]
**PG**	0.88	8855	DPP-IV inhibitor	N/A	172.169	[47]
**PG**	0.88	3285	N/A	N/A	172.169	[50]
**MR**	0.85	8836	DPP-IV inhibitor	N/A	305.386	[47]
**PL**	0.81	7513	ACE-I inhibitor	337.32 μM	228.277	[51]
**PL**	0.81	8638	DPP-IV inhibitor	N/A	228.277	[52]
**PLG**	0.8	7510	ACE-I inhibitor	4.74 μM	285.329	[51]
**PR**	0.79	3537	ACE-I inhibitor	4.10 μM	271.305	[53]
**RG**	0.74	8882	DPP-IV inhibitor	N/A	231.24	[47]
**LG**	0.72	7619	ACE-I inhibitor	8800 μM	188.212	[46]
**MA**	0.69	3173	DPP-IV inhibitor	N/A	220.277	[49]
**RL**	0.63	8886	DPP-IV inhibitor	N/A	287.348	[47]

**Table 4:** Selected peptides and predicted solubility, resistance to digestion, toxicity, and allergenicity for chosen oat peptides.

Peptide	Solubility in Water	Resistance to Digestion	Toxicity	Allergenicity Probability
FFG	Poor	No	Non-toxin	Non-allergen
IFFFL	Poor	No	Non-toxin	Non-allergen
PFL	Poor	No	Non-toxin	Non-allergen
WWK	Good	No	Non-toxin	33.3%
WCY	Poor	No	Non-toxin	33.3%
FPIL	Poor	No	Non-toxin	Non-allergen
CPA	Poor	Yes	Non-toxin	Non-allergen
FLLA	Poor	No	Non-toxin	Non-allergen
FEPL	Good	No	Non-toxin	Non-allergen

## Appendix

**Table A1:** Top 15 novel peptide hydrolysates identified following in silico hydrolysis of oat proteins with proteases papain and ficin that were predicted to have high bioactivity for the inhibition of ACE-I and DPP-IV.

Peptide	Predicted Activity	Peptide Ranker Score	Protein	Enzyme	Location	Mass (Da)
FFG *	ACE-I	1	Avenin	Papain, Ficin	f(109–111)	369.45
IFFFL *	ACE-I	0.99	Avenin	Ficin	f(3–7)	685.93
WCY *	ACE-I	0.98	11S globulin/12S globulin	Papain, Ficin/Papain	f(172–174)/f(172–174)	470.57
PFL *	ACE-I	0.98	Avenin-3/Avenin-E	Ficin/Ficin	f(84–86)/f(70–72)	375.5
WWK *	ACE-I	0.98	Avenin/Tryptophanins	Papain, Ficin/Papain, Ficin	f(70–72)/f(65–67)	518.65
IFFFLA	ACE-I	0.95	Avenin	Papain	f(3–8)	757.02
FPIL *	DPP-IV, ACE-I	0.92	11S globulin	Ficin	f(364–367)	488.68
IWR	ACE-I	0.91	Avenin/Tryptophanins	Papain/Papain	f(98–100)/f(94–96)	473.61
FPTL	DPP-IV,	0.84	12S globulin	Ficin	f(356–359)	476.62
PFV	DPP	0.82	12S globulin	Ficin	f(264–266)	361.47
CPA *	DPP-IV	0.79	11S globulin/12S globulin/Avenin-3/Avenin-E	Papain, Ficin/Papain, Ficin/Papain/Papain	f(121–123)/f(121–123)/f(116–118)/f(102–104)	289.37
FLLA *	DPP	0.79	11S globulin/12S globulin	Papain/Papain	f(203–206)/f(203–206)	462.62
FEPL *	ACE-I	0.75	12S globulin	Papain	f(53–56)	504.63
PLR	ACE-I	0.74	11S globulin/12S globulin	Papain/Papain	f(55–57)	384.51

* Peptides that were chosen for chemical synthesis and tested with in vitro assays.
